# Supplementary material for: Adeno-associated virus capsid assembly is divergent and stochastic
Source: Nat Commun. 2021 Mar 12;12:1642. doi: 10.1038/s41467-021-21935-5 (PMC7955066; doi:10.1038/s41467-021-21935-5)
Supplement: Supplementary file 1 — Supplementary Information [file 41467_2021_21935_MOESM1_ESM.pdf]

Supplementary information to:

Adeno-associated virus capsid assembly is divergent and stochastic

Tobias P. Wörner[1,2], Antonette Bennett[3], Sana Habka[1,2], Joost Snijder[1,2], Olga Friese[4], Thomas Powers[4], Mavis Agbandje-McKenna[3], Albert J.R. Heck[1,2,#]

Affiliations:

[1] Biomolecular Mass Spectrometry and Proteomics, Bijvoet Center for Biomolecular Research and Utrecht Institute for Pharmaceutical Sciences, University of Utrecht, Padualaan 8, 3584 CH Utrecht, The Netherlands;

[2] Netherlands Proteomics Center, Padualaan 8, 3584 CH Utrecht, The Netherlands;

[3] Department of Biochemistry and Molecular Biology, Center for Structural Biology, the McKnight Brain Institute, 1200 Newell Drive, Gainesville, FL 32610, USA;

[4] Biotherapeutics Pharmaceutical Sciences, Pfizer WRDM, St Louis, MO, USA;

# Corresponding Author email address: A.J.R.Heck@uu.nl

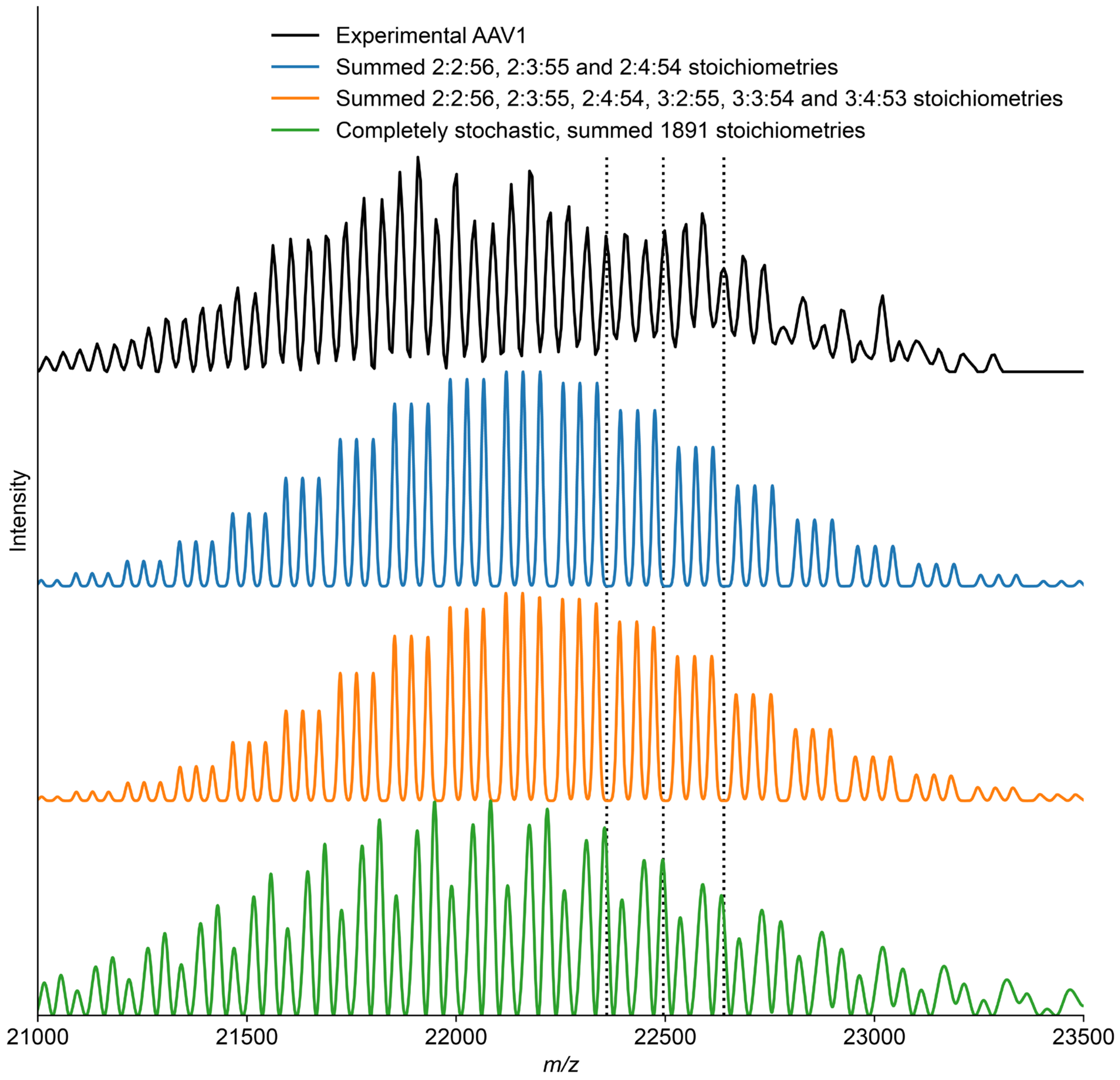

Supplemental Figure 1 - Defined vs. stochastic assembly model: Comparison between (from top to bottom) the experimental AAV1 spectrum, a simulation using only three stoichiometries based on direct assignments, a simulation with six stoichiometries based on direct assignment and potential overlap due to VP3 toVP1 substitutions, and a simulation of all possible 1891 stoichiometries with abundances following a multinomial distribution. Dotted lines indicate regions where the extended stochastic model provides a better match than the heterogeneity earlier described (27).

a

3.7 MDa

z	m/z_c	σ	I_c
147	25,171	9.1	0.3
148	25,001	9.0	0.6
149	24,833	8.9	0.8
150	24,668	8.8	1.0
151	24,504	8.7	0.8
152	24,343	8.6	0.6
153	24,184	8.5	0.3

$$I(m/z) = \frac{I_c}{\sigma\sqrt{2\pi}} e^{-\frac{1}{2}\left(\frac{m/z - m/z_c}{\sigma}\right)^2}$$

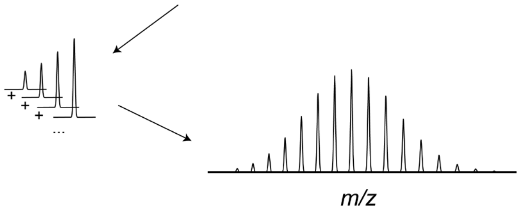

b

8:8:84 (VP1%:VP2%:VP3%)

VP1:VP2:VP3	Mass	p	z_average
0:0:60	3,568,500	2.86×10^{-5}	147
0:1:59	3,575,118	1.64×10^{-4}	147
1:0:59	3,590,269	1.64×10^{-4}	147
1:1:58	3,596,887	9.19×10^{-4}	148
0:2:58	3,581,736	4.60×10^{-4}	147
2:0:58	3,625,274	6.89×10^{-3}	148

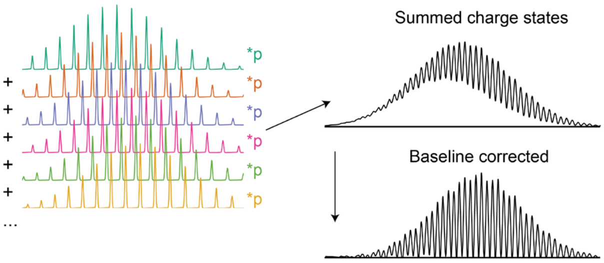

c

VP1%:VP2%:VP3%	Average Mass
0:0:100	3,568,500
0:1:99	3,572,471
1:0:99	3,581,561
1:1:98	3,585,532

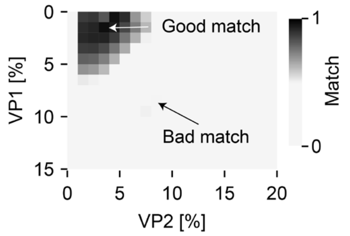

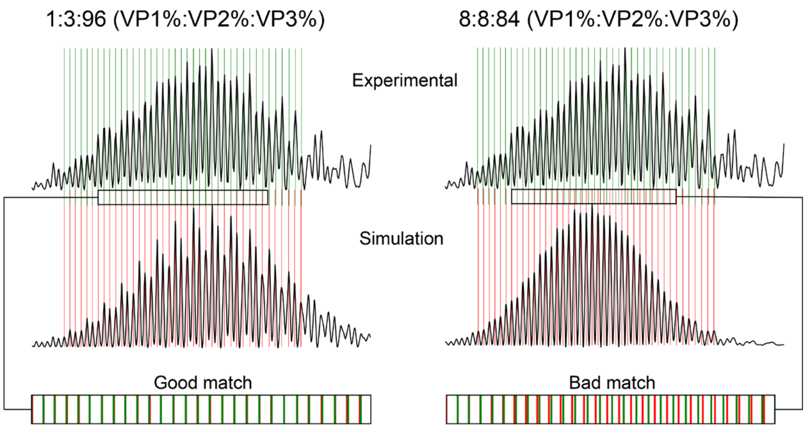

Supplemental Figure 2 - Overview of simulation and scoring workflows: a) Illustration of procedure for simulation of individual charge state distributions. For each charge state we calculated the corresponding *m/z*-position, resolution dependent peak width, and normally distributed intensity values. Using the displayed formula, each peak was calculated separately and combined to the final charge state distribution. **b**) For the Simulations of whole AAV populations we first calculated the mass distribution for all 1891 possible VP stoichiometries as shown in Figure 4a. With the calculated average charge and a fixed charge state width we calculated each charge state distribution as described in **a)** and multiply them by their probability before combining them into one final mass spectrum. For better comparability with transient averaged mass spectra we performed a baseline correction step. **c)** For the systematic comparison between simulations and experimental data we changed the bulk VP% expression levels by increments of 1%. From the average mass we adjusted the charging so the simulated charge state distribution populates the same *m/z* regions as the experimentally recorded spectrum. The peak positions are then compared and scored as described in the method section. The left spectra illustrate a good match between simulation and experimental data whereas the right spectra is an example of a bad match with diverging peaks positions.
