# Supplementary material for: Adeno-associated virus capsid assembly is divergent and stochastic
Source: Nat Commun. 2021 Mar 12;12:1642. doi: 10.1038/s41467-021-21935-5 (PMC7955066; doi:10.1038/s41467-021-21935-5)
Supplement: Supplementary file 3 — Description of Additional Supplementary Files [file 41467_2021_21935_MOESM3_ESM.docx]

**Description of Additional Supplementary Files**

File Name: Supplementary Software 1 – Code Example

Description: Python script for the simulation and scoring of complex AAV mass spectra
