# Supplementary material for: Adeno-associated virus capsid assembly is divergent and stochastic
Source: Nat Commun. 2021 Mar 12;12:1642. doi: 10.1038/s41467-021-21935-5 (PMC7955066; doi:10.1038/s41467-021-21935-5)
Supplement: Supplementary file 4 — Supplementary Software [file 41467_2021_21935_MOESM4_ESM.zip › Example_final.html]

Example\_final


# README¶

The following Jupyter notebook gives an example of AAV spectra simulation and matching as described in the method section of the manuscript. The code was tested with Pyhton 3.7.6 and the following package versions:

- scipy 1.4.1
- pandas 1.0.3
- numpy 1.18.1
- matplotlib 3.1.3

We recommend installation of python (and the required packages) as well as execution of the notebook via Anaconda3.

The Code is divided in the following sections:

1. Here we import required libraries and define functions and classes for the later spectra simulation and matching.
2. The first step is the calculation of the expected masses for all possible VP combinations and the respective probabilities/abundances following a multinomial distribution.
3. Correction of charging so simulations occupy the same m/z region as experimental spectra.
4. Simulation of mass spectrum.
5. Comparison of peak positions of simulation and experimental spectra followed by scoring.

# 0. Import of packaged and functions for script¶

In [1]:

```
#Import of required packages
from scipy.stats import multinomial, norm
from scipy.ndimage import gaussian_filter1d
from scipy.interpolate import UnivariateSpline
from scipy.signal import find_peaks
from scipy import sparse
from scipy.sparse.linalg import spsolve

import pandas as pd
import numpy as np
import matplotlib.pyplot as plt
```

In [2]:

```
# required functions and classes
def simulate_distribution(total, probs, masses):
    """
    Function calculating the probability and mass of all 1891 possible AAV stoichiometries
    """
    masses = np.array(masses) 
    probs = np.array(probs) 
    probs = probs/probs.sum()
    dist = multinomial(total, probs) # create multinominal object from pythons scipy
    combinations = [[i, j, total-i-j] for i in range(total+1) for j in range(total+1-i)] # calculate all possible VP stoichiometries
    data = pd.DataFrame({"Stoichiometry" : combinations}) # create dataframe for all stoichiometries
    data["Mass"] = data["Stoichiometry"].apply(lambda x: x[0]*masses[0]+x[1]*masses[1]+x[2]*masses[2]) # calculate mass for each stoichiometries
    data["p"] = data["Stoichiometry"].apply(lambda x: dist.pmf(x)) # calculate probability for each stoichiometries following a multinominal distribution
    return data # return DataFrame with masses and probabilities 


def baseline_als_optimized(y, lam, p, niter=10):
    L = len(y)
    D = sparse.diags([1,-2,1],[0,-1,-2], shape=(L,L-2))
    D = lam * D.dot(D.transpose()) # Precompute this term since it does not depend on `w`
    w = np.ones(L)
    W = sparse.spdiags(w, 0, L, L)
    for i in range(niter):
        W.setdiag(w) # Do not create a new matrix, just update diagonal values
        Z = W + D
        z = spsolve(Z, w*y)
        w = p * (y > z) + (1-p) * (y < z)
    return z


class Simulation():
    """
    Class doing simulation of mass spectra for Orbitrap data
    """
    def __init__(self, masses, ratios, resolution=7000, xlim=(2000, 80000), charging="auto"):
        self.masses = np.array(masses)
        self.ratios = np.array(ratios)
        self.resolution = resolution
        self.xlim = xlim
        self.charging = charging
        self.cs_width = 5
        self.cs_offset = 0
        self.dpd = 0.1
        self.ions = pd.DataFrame()


    def make_empty_spectra(self):
        self.mz = np.arange(self.xlim[0], self.xlim[1] + self.dpd, self.dpd)
        self.intensity = np.zeros(len(self.mz))

    def calculate_ions(self):
        for m, r in zip(self.masses, self.ratios):
            z_average = int(np.round(1.638*(m/1000)**0.5497)) + self.cs_offset
            z_range = range(z_average - self.cs_width, z_average + self.cs_width + 1)
            mz = [(m + z) / z for z in z_range]
            i = r * norm.pdf(np.linspace(-3,3,len(mz)))
            self.ions = self.ions.append(pd.DataFrame({"Mass" : m, "z" : z_range, "m/z" : mz, "Intensity" : i}))


    def set_resolution(self):
        A = self.resolution/400**-0.5
        self.ions["Resolution"] = A * self.ions["m/z"] ** -0.5
        self.ions["FWHM"] =  self.ions["m/z"] / self.ions["Resolution"]
        self.ions["sigma_dpd"] = self.ions["FWHM"] / 2.355 / self.dpd
        self.ions["peak_width_dpd"] = np.ceil(self.ions["sigma_dpd"] * 8)
        self.ions["m/z_rounded"] =  np.round(self.ions["m/z"] / self.dpd) * self.dpd
        self.ions["idx"] = (self.mz2idx(self.ions["m/z_rounded"], self.xlim, self.dpd)).astype(int)
        self.ions = self.ions[self.ions["m/z_rounded"].between(self.xlim[0], self.xlim[1])]


    def make_trace(self):
        self.final_idx = []
        self.final_intensity = []
        self.ions[["Intensity", "sigma_dpd", "peak_width_dpd", "idx"]].apply(tuple, 1).apply(lambda x: self.convolute(x[0], x[1], x[2], x[3]))
        ions = pd.DataFrame({"idx" : np.concatenate(self.final_idx), "Intensity" : np.concatenate(self.final_intensity)})
        ions = ions.groupby("idx").sum().reset_index()
        ions["idx"] = ions["idx"].astype(int)
        self.intensity[ions["idx"]] = ions["Intensity"]
        self.intensity = self.intensity / self.intensity.max()

    def mz2idx(self, mz, xlim, dpd):
        rel_mz = mz - xlim[0]
        idx = rel_mz / dpd
        return idx

    def convolute(self, intensity, sigma, dist_range, idx):
        self.final_idx.append(np.arange(idx - round(dist_range/2), idx + 1 + round(dist_range/2)))
        self.final_intensity.append(intensity * norm.pdf(np.arange( -round(dist_range/2),round(dist_range/2) + 1, 1), scale = sigma))


def match_peaks(peaks_sim, peaks_trace, mean_peak_dist):
    peaks_sim = list(peaks_sim)
    matched_peaks = pd.DataFrame({"trace" : peaks_trace, "sim" : np.NaN})
    for idx, row in matched_peaks.iterrows():
        if len(peaks_sim) > 1:
            idx_closest = np.argmin(np.abs(row["trace"] - peaks_sim))
            if np.abs(row["trace"] - peaks_sim[idx_closest]) < mean_peak_dist / 2:
                mz = peaks_sim.pop(idx_closest)
                matched_peaks.loc[idx,"sim"] = mz
    unmached_peak_dict = np.array(peaks_sim)
    return matched_peaks, unmached_peak_dict


def score(peak_df, unmached_peak, lim=None):
    if lim:
        peak_df = peak_df[peak_df["trace"].between(lim[0], lim[1])]
    spectra = peak_df["trace"]
    sim = peak_df["sim"]
    
    return (np.mean((spectra-sim)**2)**0.5 + sim.isna().sum() + np.sum((unmached_peak > lim[0]) & (unmached_peak < lim[1])))
```

# 1. Simulation of AAV mass distribution following stochastic assembly mechanism¶

In [3]:

```
####################################################################################
# Calculation of AAV mass distribution
####################################################################################


#Defining some parameters
masses = [81625, 66519, 59763] # Masses of VP1,VP2, and VP3 in Da
prob = [1, 3, 96] # bulk VP expression ratios


solvent = 2800 #solvent adducts in Da


distribution = simulate_distribution(60, prob, masses)
distribution["Mass_w_solvent"] = distribution["Mass"] + solvent


fig, ax = plt.subplots(figsize=(10, 5))
plt.hist(distribution["Mass_w_solvent"]/1e3, weights=distribution["p"],bins=500)
plt.xlabel("Mass [kDa]")
plt.ylabel("Probability");
```

# 2. Charge correction for simulations¶

In [4]:

```
####################################################################################
# Correction of charging so simulated mass spectra always populate the same m/z region as experimental data
####################################################################################


average_mz = 22300 #m/z region of experimental spectrum

#calculatin of expected average charge without correction
centroid_mass = (distribution["Mass"] * distribution["p"]).sum() / distribution["p"].sum()
epxpected_z = 1.638*(centroid_mass/1000)**0.5497
epxpected_z
#average charge so it populates the experimental  m/z region
measured_z = centroid_mass/average_mz
measured_z

#calculate offset to be used in simulation from normal charging
cs_offset = np.round(measured_z - epxpected_z).astype(int)
print("A charge offset of %d z has to be applied so the simulated mass spectra occupies the same m/z region as the measured spectra at %d m/z"%(cs_offset, average_mz))
```

```
A charge offset of 14 z has to be applied so the simulated mass spectra occupies the same m/z region as the measured spectra at 22300 m/z
```

# 3. Simulation of AAV mass spectra¶

In [5]:

```
####################################################################################
#AAV mass spectrum simulation
####################################################################################


#Defining some parameters
res = 12000 #resolution setting
cs_width = 12 # charge states per mass


# Here we do the actual simulation of the mass spectrum with the function defined in 0. and with the masses and probabilities in 1.
AAV_sym = Simulation(distribution["Mass_w_solvent"], distribution["p"], resolution=res)
AAV_sym.xlim = (10000, 40000)
AAV_sym.cs_offset = cs_offset # Here we apply the calculated charging offset from 2.
AAV_sym.cs_width = cs_width
AAV_sym.dpd = 1

AAV_sym.make_empty_spectra()
AAV_sym.calculate_ions()
AAV_sym.set_resolution()
AAV_sym.make_trace()


#Visualiazation of spectra and show effect of baseline correction
fig, ax = plt.subplots(figsize=(10, 5))


plt.plot(AAV_sym.mz, AAV_sym.intensity+1, label="before baseline correction")


z = baseline_als_optimized(AAV_sym.intensity, 3000000, 0.0005)
AAV_basleine_corrected = AAV_sym.intensity - z
AAV_basleine_corrected = AAV_basleine_corrected / AAV_basleine_corrected.max()

plt.plot(AAV_sym.mz, AAV_basleine_corrected, label="after baseline correction")
plt.legend()
plt.xlim(20000, 24000)
ax.set_yticks([])
plt.xlabel("m/z");
```

# 4. Spectra matching and scoring¶

In [6]:

```
####################################################################################
#Comparison between experimental data and simulation with peak matching and scoring#
####################################################################################


# This section loads and extracts the peaks of the experimental spectrum
trace = pd.read_csv("AAV8_1.txt", sep="\t", names=["m/z", "Intensity"])
trace["Intensity"] = trace["Intensity"] / trace["Intensity"].max()


# This section extracts the peaks of the experimental spectrum
spl = UnivariateSpline(trace["m/z"], trace["Intensity"], k=4,  s=0) #SPL interpolation to compensate for low number of datapoints
xx = np.arange(19000, 24000, 1)
yy = spl(xx)
# a bit of smoothing
smooth_window = 1
smooth_cycles = 10
while smooth_cycles != 0:
    yy = gaussian_filter1d(yy, sigma=smooth_window)
    smooth_cycles -= 1
# Extraction of peak positions for experimental spectra
peaks_idx, peak_height = find_peaks(yy, height=0.01)
peaks_trace = xx[peaks_idx]


# This section extracts the peaks of the simulated spectrum
xx = AAV_sym.mz
yy = AAV_sym.intensity


smooth_window = 1
smooth_cycles = 10
while smooth_cycles != 0:
    yy = gaussian_filter1d(yy, sigma=smooth_window)
    smooth_cycles -= 1

peaks_idx, peak_height = find_peaks(yy, height=0.01)
peaks_sim = xx[peaks_idx]


#matching the peaks from experimental and simulation with each other, 45 Th is the average distance between the peaks
matched_peaks, unmached_peak_dict = match_peaks(peaks_sim, peaks_trace, 45)
#scoring the matched peak distances
score_label = score(matched_peaks, unmached_peak_dict, lim=[21200,23000])


#plotting the two spectra and matched peak position as dotted lines 

fig, ax = plt.subplots(figsize=(10, 5))
plt.plot(trace["m/z"], trace["Intensity"]+1, label="Experimental")

plt.plot(AAV_sym.mz, AAV_sym.intensity, label="Simulation")

filtered_matched_peaks = matched_peaks[matched_peaks["trace"].between(21200,23000)]
[plt.plot([mz, mz], [0,1], color="k", ls=":") for mz in filtered_matched_peaks["sim"]]
[plt.plot([mz, mz], [1,2], color="k", ls=":") for mz in filtered_matched_peaks["trace"]]


plt.legend()
plt.xlim(20500, 24000)
ax.set_yticks([])
plt.xlabel("m/z")
plt.title("%.3f Th average deviation"%score_label);
```
